# Expression of the cobalamin transporters cubam and MRP1 in the canine ileum–Upregulation in chronic inflammatory enteropathy

**DOI:** 10.1371/journal.pone.0296024

**Published:** 2024-01-11

**Authors:** Stefanie Kather, Johannes Kacza, Helga Pfannkuche, Denny Böttcher, Chi-Hsuan Sung, Joerg M. Steiner, Gotthold Gäbel, Franziska Dengler, Romy M. Heilmann

**Affiliations:** 1 Small Animal Clinic, Veterinary Teaching Hospital, College of Veterinary Medicine, University of Leipzig, Leipzig, SN, Germany; 2 Institute of Veterinary Physiology, College of Veterinary Medicine, University of Leipzig, Leipzig, SN, Germany; 3 BioImaging Core Facility, College of Veterinary Medicine, Saxon Incubator for Clinical Translation, University of Leipzig, Leipzig, SN, Germany; 4 Institute of Veterinary Pathology, College of Veterinary Medicine, University of Leipzig, Leipzig, SN, Germany; 5 Gastrointestinal Laboratory, School of Veterinary Medicine and Biomedical Sciences, Texas A&M University, College Station, TX, United States of America; 6 Institute for Physiology, Pathophysiology and Biophysics, University of Veterinary Medicine, Vienna, Austria; Bauer Research Foundation, UNITED STATES

## Abstract

Chronic inflammatory enteropathy (CIE) in dogs, a spontaneous model of human inflammatory bowel disease (IBD), is associated with a high rate of cobalamin deficiency. The etiology of hypocobalaminemia in human IBD and canine CIE remains unknown, and compromised intestinal uptake of cobalamin resulting from ileal cobalamin receptor deficiency has been proposed as a possible cause. Here, we evaluated the intestinal expression of the cobalamin receptor subunits, amnionless (AMN) and cubilin (CUBN), and the basolateral efflux transporter multi-drug resistance protein 1 (MRP1) in 22 dogs with CIE in comparison to healthy dogs. Epithelial CUBN and AMN levels were quantified by confocal laser scanning microscopy using immunohistochemistry in endoscopic ileal biopsies from dogs with (i) CIE and normocobalaminemia, (ii) CIE and suboptimal serum cobalamin status, (iii) CIE and severe hypocobalaminemia, and (iv) healthy controls. CUBN and MRP1 expression was quantified by RT-qPCR. Receptor expression was evaluated for correlation with clinical patient data. Ileal mucosal protein levels of AMN and CUBN as well as mRNA levels of CUBN and MRP1 were significantly increased in dogs with CIE compared to healthy controls. Ileal cobalamin receptor expression was positively correlated with age, clinical disease activity index (CCECAI) score, and lacteal dilation in the ileum, inversely correlated with serum folate concentrations, but was not associated with serum cobalamin concentrations. Cobalamin receptor downregulation does not appear to be the primary cause of hypocobalaminemia in canine CIE. In dogs of older age with severe clinical signs and/or microscopic intestinal lesions, intestinal cobalamin receptor upregulation is proposed as a mechanism to compensate for CIE-associated hypocobalaminemia. These results support oral supplementation strategies in hypocobalaminemic CIE patients.

## Introduction

Inflammatory bowel disease (IBD) is a common cause of gastrointestinal (GI) signs with multifactorial pathogenesis in humans and companion animals and significantly reduces the quality of life of affected patients [1]. The term IBD refers to mostly Crohn’s disease and ulcerative colitis in people [2], and the incidence of IBD has been increasing since the beginning of the 21^st^ century [3]. IBD is currently one of the most prevalent GI diseases in Europe and North America, and an increase is observed in newly industrialized countries of Asia, Africa, and South America [4,5]. Dogs have been sharing the environment with humans for thousands of years and also share many civilization diseases, such as IBD. While there are many differences in clinical and histopathologic features of IBD between humans and dogs (e.g., a more multifocal distribution along the small intestine), some aspects of pathogenesis are shared, and the disease in dogs has been proposed as a spontaneous model for further understanding the pathogenesis of IBD in humans [6]. IBD in humans and dogs cause similar clinical signs such as diarrhea, vomiting, and weight loss that persist or chronically recur [6]. IBD is currently referred to as chronic inflammatory enteropathy (CIE) in dogs and can be subclassified into food-responsive enteropathy (FRE) and antibiotic-responsive enteropathy (ARE, though the existence of this entity has recently been questioned [7,8]), immunosuppressant-responsive, or non-responsive IBD based on the response to treatment trials [7–12].

Hypocobalaminemia is highly prevalent in dogs with CIE, particularly immunosuppressant-responsive and non-responsive IBD, ranging from 19–54% [10,13,14]. In human IBD, the prevalence of cobalamin deficiency is lower (10–20%) but varies with disease location and appears to be related to prior ileocolonic resection [15,16]. Cobalamin (vitamin B_12_) is needed for many intracellular functions and mucosal regeneration [17]. As an essential cofactor for the intracellular enzymes cytoplasmatic methionine synthase and mitochondrial methylmalonyl-CoA mutase [18], cobalamin deficiency can lead to functional folate deficiency and increased serum concentrations of homocysteine (HCY), both due to methionine synthase inactivation, and accumulation of methylmalonic acid (MMA) due to reduced methylmalonyl CoA mutase activity [19–21]. Hence, cobalamin deficiency can be associated with neurologic disorders due to increased intracellular and systemic MMA concentrations and hyperammonemia [22–24]. Blood cell count abnormalities are not typically present in dogs [25,26] but can be an early sign of imbalances in cobalamin metabolism in people (e.g., macrocytic anemia and leucopenia) [17]. With progression, disorders of phospholipid and amino acid metabolism occur in both species [17,27,28]. Additionally, hypocobalaminemic dogs often do not respond to treatment for CIE, unless given supplemental cobalamin [29]. In cases of severe cobalamin deficiency, clinical signs such as vomiting, diarrhea, anorexia, and lethargy can occur.

Adequate cobalamin serum levels and cellular supply depend upon efficient intestinal uptake. Cobalamin is mainly ingested with food of animal origin and bound to salivary haptocorrin in the stomach [30]. Reaching the duodenum, cobalamin is bound to intrinsic factor (IF), which in dogs is produced primarily by the exocrine pancreas [31–33]. The intestinal cobalamin-IF receptor, known as cubam, is localized at the brush border of the ileal epithelium and is comprised of two subunits, the proteins cubilin (CUBN) as the primary binding site for the IF-cobalamin receptor complex and amnionless (AMN), which internalizes the receptor into enterocytes [33,34]. The cobalamin-IF-complex is absorbed into the enterocyte by receptor-mediated endocytosis [31]. Cobalamin is then separated from the receptor and IF within the endolysosome and is transported across the basolateral cell membrane, either in its free form or bound to transcobalamin II, by multidrug resistance protein 1 (MRP1) [35]. However, parts of the transport within polarized epithelial cells (e.g., enterocytes), and specifically within the basolateral compartment, are still unknown. A role of megalin in cobalamin trafficking was assumed by the colocalization of cubilin with megalin in several epithelia [36], but the terminal ileum in humans lacks megalin [37]. In plasma, cobalamin is bound to the transport proteins transcobalamin II (binding biologically active cobalamin, which is available to the cells) or haptocorrin (binding biologically inactive cobalamin, which is not available to the cells) [38,39].

Hypocobalaminemia is associated with increased intraepithelial lymphocyte counts and histologic lesion severity in the ileum [40,41], suggesting a possible link between ileal mucosal inflammation and cobalamin malabsorption in canine CIE. It was proposed that inflammation-induced reduction in the ileal cobalamin receptor expression leads to impaired absorption of cobalamin, causing hypocobalaminemia or cobalamin deficiency in CIE [42]. Significant changes in intestinal mucosal gene and protein expression profiles are reported in IBD in people [43–45] and CIE in dogs [46]. As an example, proinflammatory cytokines, such as tumor necrosis factor-alpha (*TNFA*), were shown to be increased in people with IBD [47] and dogs with CIE [48]. Cyclooxygenase-2 (*COX-2*), as a key enzyme for the synthesis of proinflammatory cytokines, was also upregulated in some dogs with IBD [49].

However, the intestinal expression of the cobalamin receptor and the possibility of dysregulated intestinal cobalamin receptor expression in patients with IBD (or dogs with CIE) have not yet been evaluated. A better understanding of the pathophysiology of cobalamin deficiency in CIE will ultimately allow optimizing the individually tailored treatment strategy in affected dogs and, from a translational aspect, provide further insight into the pathophysiology of cobalamin deficiency in humans with IBD.

Thus, our study aimed to quantify the abundance of the proteins involved in intestinal cobalamin uptake in ileal biopsies from dogs diagnosed with CIE compared to healthy dogs and evaluate those receptor levels in relation to patient clinical, clinicopathologic, and histologic variables.

## Materials and methods

### Animals

#### Ethics approval

The enrollment of dogs with CIE in the study of several biomarkers was approved by the Clinical Research Review Committee at Texas A&M University (CRRC approval #TAMU 2009–06, approved 01-15-2009), the Institutional Animal Care and Use Committee at Texas A&M University (IACUC approval #TAMU 2012–083, approved 05-22-2012), and the Regional Council of the State of Saxony, Chemnitz/Leipzig, Germany (Animal Use Protocol #TVV 06–17, approved 04-11-2017). Control tissue biopsies were obtained from purpose-bred healthy dogs euthanized for an unrelated project at the School of Veterinary Medicine and Biomedical Sciences at Texas A&M University (Animal Use Protocol #TAMU 2009–0123).

#### Healthy controls

Ileal full-thickness tissue biopsies obtained from healthy dogs (n = 9 for confocal laser scanning microscopy, n = 11 for mRNA analysis) were used for this study. To be included as healthy control, the dogs could not have any clinical signs of GI disease (e.g., vomiting, abnormal stool consistency and/or frequency, weight loss, inappetence, reduced attitude/activity, ascites, and/or pruritus) or receive any medication known to affect the GI tract and had to be regularly vaccinated and dewormed.

#### Dogs with CIE

For immunofluorescence analysis, 22 dogs diagnosed with CIE were included (Table 1). Endoscopic tissue biopsies of the ileum, whole blood, and serum samples were collected from these dogs between May 2011 and November 2019 at the Veterinary Teaching Hospital at Texas A&M University, TX and the University of Leipzig Small Animal Clinic (UL-SAC), SN, Germany. For RT-qPCR, endoscopic tissue biopsies of the ileum were collected from 5 dogs presented to the UL-SAC between December 2018 and December 2020 for an unrelated study [50]. Dogs were included if they showed clinical signs of chronic enteropathy (i.e., vomiting, diarrhea, and/or weight loss for ≥3 weeks) and other possible causes of chronic GI signs (e.g., atypical hypoadrenocorticism with a similar clinical presentation as CIE or exocrine pancreatic insufficiency as IF is primarily of pancreatic origin in dogs and affected dogs have a reduced cobalamin release from haptocorrin and binding to IF as a consequence of deficient exocrine pancreatic secretion) were excluded. Intestinal inflammation was documented histologically, and the response to treatment supported the diagnosis of CIE [6,12]. Cobalamin could not have been supplemented in these patients, and no anti-inflammatory and/or immunosuppressive medication administered within 4 weeks prior to enrollment into the study. There were no restrictions on breed, sex, or age for inclusion into the study.

**Table 1:** Characteristics of all dogs with CIE (n = 22) included in the confocal laser scanning microscopy analysis.

Patient characteristic	Normocobalaminemia	Suboptimal serum cobalamin	Hypocobalaminemia
n	9	8	5
Age, in years	7.8 (1.3–11.0)^A,B^	**6.1 (0.5–9.3)** ^**A**^	**10.0 (5.3–11.9)** ^**B**^
Sex, male (neutered) / female (spayed)	6 (3) / 3 (3)	4 (3) / 4 (3)	3 (3) / 2 (2)
Body weight, in kg	15.2 (3.6–38.3)	9.6 (3.0–31.0)	28.1 (9.5–34.9)
***Clinical parameters***			
CCECAI score	5 (1–12)	7 (2–20)	7 (4–12)
***Clinicopathologic parameters***			
Serum cobalamin, in ng/L	**647 (425–1000)** ^**A**^	**305 (259–367)** ^**B**^	**186 (132–208)** ^**C**^
Serum folate, in μg/L	10.8 (9.4–25.8)^A^	15.0 (11.4–21.5)^A^	**8.3 (6.2–11.6)** ^**B**^
Serum albumin, in g/L	29 (9–43)	28 (11–34)	23 (12–28)
Serum CRP, in mg/L	10.0 (0.1–30.6)^†^	21.3 (0.8–59.8)^$^	60.0 (14.5–60.0)^‡^
***Histopathologic parameters***			
Duodenum, composite score	1 (1–2)	2 (1–2)	2 (1–2)
Morphologic criteria			
• Villus stunting	0 (0–2)	0.5 (0–1)	1 (0–1)
• Epithelial injury	0 (0–1)	0 (0)	0 (0–0.5)
• Crypt distension	0 (0–2)	0 (0–2)	0.5 (0–1)
• Lacteal dilation	0 (0–2)	0.25 (0–2)	1 (0.5–1)
• Mucosal fibrosis	0 (0–2)	0 (0)	0 (0–1)
Inflammatory criteria			
• IEL	1 (0–2)	0 (0–1)	0.5 (0.5–1)
• LP LPC	1 (1–2)	2 (0–2)	2 (1.5–2)
• LP eosinophils	1 (0–2)	1 (0–2)	0.5 (0–1)
• LP neutrophils	0 (0–1)	0 (0–1)	0.5 (0–1)
• LP ΜΦ	0 (0–1)	0 (0–1)	1 (0.5–1)
Ileum, composite score	1 (1–2)	1 (1–2)	2 (1–2.5)
Morphologic criteria			
• Villus stunting	0 (0–2)	1 (0–2)	1 (0–1)
• Epithelial injury	0 (0)	0 (0–3)	0 (0–1)
• Crypt distension	0 (0–2)	0 (0–3)	0.5 (0–2)
• Lacteal dilation	0 (0–3)	0 (0–2.5)	0 (0–1)
• Mucosal fibrosis	0 (0–1)	0 (0)	0 (0–1)
Inflammatory criteria			
• IEL	0 (0–2)	0 (0–2)	0.5 (0–1)
• LP LPC	1 (0–2)	1 (0–2)	1 (0–2)
• LP eosinophils	1 (0–2)	0.5 (0–1)	0 (0–1)
• LP neutrophils	0 (0–1)	0 (0–1)	0 (0–2)
• LP ΜΦ	0 (0–1)	0 (0–1)	0.5 (0–3)

CCECAI: Canine chronic enteropathy clinical activity index; CRP: C-reactive protein; IEL: Intraepithelial lymphocytes; IQR: Interquartile range; LP: Lamina propria; LPC: Lymphocytes and plasma cells; ΜΦ: Macrophages. ^†^available from n = 7 dogs; ^$^available from n = 6 dogs; ^‡^available from n = 3 dogs. Parameters in bold font indicate significant differences at *P*<0.05, where values stacked within a column that are not sharing a common superscript letter and highlighted in blue are significantly different at *P*<0.05. All data are shown as medians with ranges (min-max) in parentheses. Combined upper (esophagogastroduodenoscopy) and lower GI endoscopy (ileocolonoscopy) to investigate the GI mucosa and obtain multiple GI tissue biopsies is routinely performed; data are shown only for the small intestine as the primary disease location in dogs with CIE.

Each dog underwent routine diagnostic evaluation, including a thorough patient history, complete physical examination, clinicopathologic evaluation, diagnostic imaging, and combined upper and lower GI endoscopy to obtain GI tissue biopsies. The severity of clinical disease was assessed by the attending veterinarian using the canine chronic enteropathy clinical activity index (CCECAI) [10], which considers nine parameters, each evaluated on a 3-point scale (0: normal, 1: mild changes, 2: moderate changes, 3: marked changes): attitude/activity, appetite, frequency of vomiting, stool consistency, frequency of defecation, weight loss, serum albumin concentration, peripheral edema/ascites, and pruritus. The cumulative CCECAI score is interpreted as clinically insignificant disease (cumulative score of 0–3), mild clinical disease (cumulative score of 4–5), moderate clinical disease (cumulative score of 6–8), severe clinical disease (cumulative score of 9–11), or very severe disease (score ≥12) [10].

A complete blood cell count and serum biochemistry profile, serum concentrations of cobalamin, folate, specific canine pancreatic lipase (measured as Spec cPL), trypsin-like immunoreactivity (TLI), and C-reactive protein (CRP) were measured. A urinalysis and, if indicated, quantitative urine culture and urine protein-to-creatinine ratio to evaluate for possible renal protein loss were performed.

Dogs with CIE were assigned to one of the following groups: (i) normocobalaminemia (n = 9; serum cobalamin concentration 400–908 ng/L), (ii) suboptimal serum cobalamin status (n = 8; serum cobalamin concentration 251–400 ng/L), and (iii) hypocobalaminemia (n = 5; serum cobalamin concentration ≤250 ng/L or not detectable), according to our previously proposed classification for serum cobalamin concentrations [39]. Due to the small sample size in the RT-qPCR analysis (n = 5; median serum cobalamin concentration: 258 ng/L), further stratification of this CIE group based on serum cobalamin status was not possible.

Upper and lower GI endoscopy were performed on each dog, and endoscopic tissue biopsy specimens were collected. All tissue biopsies were histologically evaluated by one of six board-certified veterinary pathologists using the current consensus guideline [51,52]. Structural and inflammatory lesions in each segment were individually graded as absent (score = 0), mild (score = 1), moderate (score = 2), or severe (score = 3), and cumulative scores calculated for the duodenum and ileum were used for statistical analyses.

### Quantification of ileal epithelial protein levels

#### Immunofluorescence staining

Tissue biopsy samples of canine ileum were fixed in 10% neutral buffered formalin, paraffin-embedded, and sectioned at 7 μm thickness. After deparaffinization, antigens were demasked with citric acid (pH 6.0, 10 mM) at 95°C. Tissue sections were then preincubated with PBS/NaN_3_-Hs-Tx (Triton^TM^ X-100 and sodium azide ReagentPlus^®^, Sigma Aldrich, Darmstadt, Germany; horse serum: c.c. pro, Oberdorla, Germany) and the tissue sections incubated with the primary antibody (1:500 polyclonal rabbit anti-AMN, ab224213, Abcam, Cambridge, UK; or 1:50 polyclonal rabbit anti-CUBN, PA5-67616, Thermo Fisher Scientific, Dreieich, Germany). After washing, the slides were incubated with a fluorescence-labeled secondary antibody (1:200 Alexa Fluor^®^ 488-AffiniPure donkey anti-rabbit IgG, 711-545-152, Dianova, Hamburg, Germany). Nuclei were counterstained with 4’,6-diamidino-2-phenylindol dihydrochloride (DAPI; Carl Roth, Karlsruhe, Germany) and coverslips mounted with glycerol gelatin (Merck, Darmstadt, Germany).

Antibody specificity was verified by incubation with the secondary antibody only and by preincubation of the primary antibody with a blocking peptide (AMN: ab139248, Abcam; CUBN: custom-designed peptide, Thermo Fisher) (S1–S3 Figs). Alignment of the amino acid (AA) sequences between the human and canine homologs (NCBI protein blast; accessed 06-20-2023) revealed an identity of 83.1% for CUBN and 77.5% for AMN (AA1–200).

#### Data acquisition and analysis

AMN and CUBN expression were documented using a confocal laser scanning microscope (CLSM; Leica TCS SP8, Leica Microsystems, Wetzlar, Germany) and the data analyzed using the Leica Application Suite X software (LAS X 3.5.5). For each dog, 1–5 (mean: 2) tissue sections of an ileal biopsy were evaluated for AMN and CUBN labeling. First, a tile scan was obtained from each tissue section using a 63x/1.30 Glyc objective (resolution 256×256-pixel, frame sequential scan at 600 Hz bidirectional, pinhole: 1 airy unit [AU]). Alexa Fluor^®^ 488 (channel 1) was excited at 488 nm (detection range: 492–600 nm) and DAPI (channel 2) at 405 nm (detection range: 407–486 nm). The overview scan served to select 5–15 (mean: 10) positions showing cross-sectioned ileal epithelial cells, which were used for analysis by photon counting. A focus map (max. intensity mode) was created to ensure subsequent analytical scanning was performed at levels of maximum signal intensity (focus level) at each selected position. At the selected positions, channel 1 was then scanned with a hybrid detector (HyD) in ‘counting mode’ (scan resolution: 512×512 pixel at 200 Hz unidirectional, pinhole: 1 AU). Transmission of the 488 nm-laser line, once set using the saturation control within the counting mode, was kept unchanged for all photon count recordings ensuring consistent scanning parameters.

**Fig 1:**
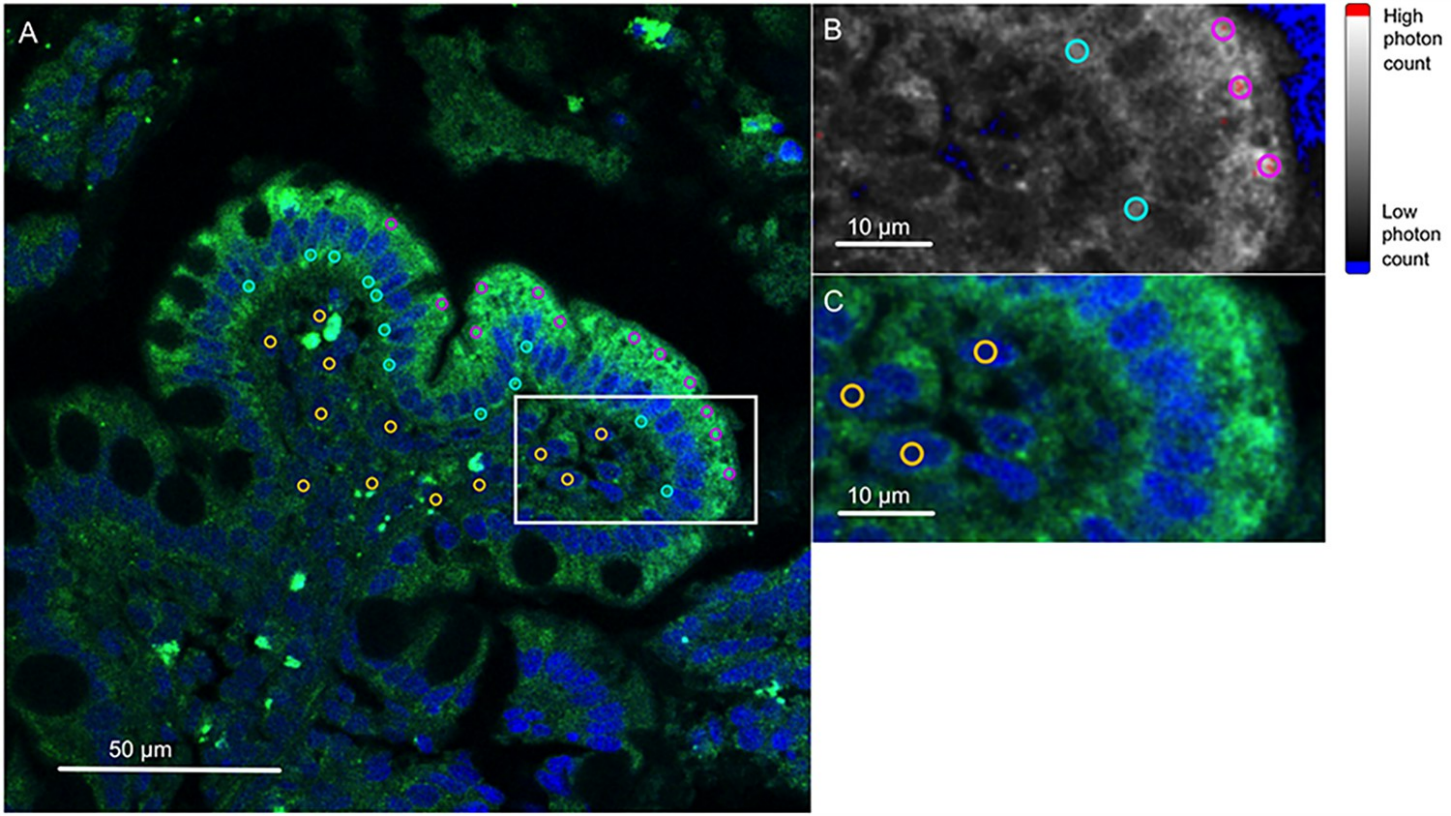
Evaluation of cobalamin receptor abundances in canine ileal epithelium–Data acquisition and image analysis via confocal laser scanning microscopy. A: Immunofluorescence staining of the amnionless (AMN) subunit (green) in the cross-sectioned ileum of a dog with chronic inflammatory enteropathy (CIE). Nuclei are DAPI (diamidine phenylindole)-stained (blue). For quantification by photon counting, 12 regions of interest (ROI) each were set in the apical (pink circles) and basolateral part (turquoise circles) of the epithelial cells, and within the nuclei of subepithelial parenchymal cells (yellow circles; controls). Focal green staining within the lamina propria is elicited by nonspecific fluorescence of erythrocytes. B: Grayscale-converted label (frame in A) showing the highest (red voxel) and lowest photon counts (blue voxel) by thresholding the saturation points of the signal. ROI were set at the sites of highest photon counts within the apical and basolateral part of the enterocytes. C: Detail (frame in A) showing ROI within the nuclei of subepithelial parenchymal cells used for data normalization.

For quantification of the CUBN and AMN expression, circular regions of interest (ROI; diameter: 2 μm, area: 3.14 μm^2^) were used to detect the maximum photon counts within the cytoplasmic compartment of the ileal epithelial cells (Fig 1A). Twelve ROI were set in the apical compartment (presumed to reflect receptors available for luminal cobalamin absorption) and within the basolateral compartment of microscopically intact epithelial cells (evaluating cobalamin sorting to that side and/or receptor recycling). The saturation control of LAS-X served to identify regions of highest photon counts within those cellular compartments, showing maximum values as red voxels (Fig 1B). Twelve control ROI were set in the area of the nuclei of subepithelial parenchymal cells, which are presumed to lack AMN and CUBN expression (Fig 1C). The average photon counts obtained from the 12 cytoplasmic ROI were then normalized against the average photon counts within the corresponding control region: [normalized epithelial photon count (nEPC) = (average photon count of the 12 epithelial ROI / average photon count of the 12 control ROI)], and the mean nEPC was calculated for each ileal tissue biopsy. The mean apical and basolateral nEPC of all biopsies from the same dog were calculated and used for statistical analyses.

### Quantification of ileal mucosal gene expression

#### mRNA extraction

Tissue samples were stored in RNAlater^®^ (Qiagen, Hilden, Germany) at -80°C until RNA isolation. Total RNA was extracted from 10 mg of tissue using the ReliaPrep^TM^ RNA Tissue Miniprep System (Promega, Walldorf, Germany). RNA quantity and quality were determined spectrophotometrically (6131 Spectrometer, Eppendorf, Hamburg, Germany) and by capillary electrophoresis (RNA 6000 Nano assay, 2100 Bioanalyzer, Agilent Technologies, Waldbronn, Germany).

#### RT-qPCR analyses

One μg of RNA was used to synthesize cDNA with the GoScript^TM^ RT System (Promega) in an MJ Research PTC-200 Peltier Thermal Cycler (Bio-Rad, Munich, Germany). For qPCR, 2 μL of the resulting cDNA were used at a 1:20 dilution in the reaction mix using the GoTaq^®^ qPCR System (Promega) and a Corbett Rotor-Gene 6000 Realtime PCR Thermocycler (Qiagen) with the following protocol: denaturation at 95°C for 5 min, followed by 45 cycles of 5-sec denaturation at 95°C, primer annealing at the optimal temperature (S1 Table) for 30 sec, and elongation at 60°C for 30 sec. Duplicate analyses were performed for each sample. If Ct values differed by >0.3, the run was discarded and repeated. Nuclease-free water instead of cDNA served as negative control in each run.

Target genes were *CUBN*, *MRP1*, and the inflammatory mediators *TNFA* and *COX-2*. Due to using full-thickness biopsies from healthy controls as opposed to endoscopic tissue biopsies from dogs with CIE, vimentin (*VIM*) was used as a marker for mesenchymal tissue content of the biopsy. Hypoxanthine-guanine phosphoribosyltransferase 1 (*HPRT1*), succinate dehydrogenase subunit A (*SDHA*), and ribosomal protein L8 (*RPL8*) were used as reference genes (S1 Table), with stability verified using RefFinder [53]. Primers were designed with the primer design tool from the National Center for Biotechnology Information (NCBI, Bethesda, MD) and produced by Eurofins Genomics (Ebersberg, Germany). Amplicons were sequenced and primer specificity verified by BLAST (basic local alignment search tool, NCBI) analysis of the sequences obtained. The quantification cycle and amplification efficiency of each primer were determined using the Rotor-Gene 6000 software v.1.7 (Corbett, Qiagen).

Mean Ct values of the target genes were normalized against the mean Ct values of the reference genes for each sample and expression levels were analyzed using the ddCt method [54]. The ratio between the target genes and *VIM* was used for data analysis to eliminate variability due to connective tissue content (ratio = [ddCt target gene/ddCt *VIM*]).

### Statistical analyses

A commercially available software package (SPSS Statistics v.21, IBM, Armonk, NY) was used for all statistical analyses. Continuous variables were tested for normality of their distribution and equality of the variances using a Shapiro-Wilk *W* test and a Brown-Forsythe test. Summary statistics for continuous variables are reported as medians and ranges, for categorical data as counts (*n*) and percentages.

A non-parametric Kruskal-Wallis test with Dunn’s post-hoc test or Mann-Whitney-*U*-test was used to compare nEPC of AMN and CUBN immunoreactivity, and the gene expression of *CUBN*, *MRP1*, *TNFA*, and *COX-2*, among the different groups or between individual groups of dogs with CIE and healthy controls. A non-parametric Spearman rank correlation coefficient *ρ* was calculated to test for possible correlations between AMN or CUBN protein levels and the patient clinical parameters (i.e., age, CCECAI score, serum cobalamin, folate, CRP, and albumin concentrations, histologic lesion severity grades for the individual criteria and cumulative lesion scores). The Spearman *ρ* was interpreted as indicating a very strong (0.8–1.0), moderate (0.6–0.8), fair (0.3–0.6), or poor (0–0.3) correlation [55]. A standard least squares model was used to identify any potential interaction between categorical variables with a significant effect on ileal AMN and/or CUBN protein levels. Statistical significance was set at *P*<0.05, and a Holm-Bonferroni correction for multiple correlations at the same level (*P*_*corr*_ = *P* ÷ n) was applied to reduce the risk of type I statistical error, if indicated. Assuming at least 2-fold differences (d_z_ = 1), which we consider the lowest threshold for biological relevance, we would thus have to test a total sample size of n = 28 to find a significant difference between the groups at α = 0.05 with a power of 0.8.

## Results

### Patient data

Dogs with CIE (n = 24) were between 0.4–11.9 years old (median: 7.5 years), represented 19 different dog breeds, and had CCECAI scores ranging from 1–12 (median: 6) (Tables 1 and S2). Histologic lesion scores in ileal biopsies ranged from 0 (no abnormalities) to 3 (severe lesions), with a median score of 1 indicating mild histologic changes.

Healthy dogs (n = 18) included in this study were 1.0–10.1 years old (median: 3.4 years), purpose-bred hound dogs, and comprised 15 females and 3 males.

### Ileal AMN and CUBN protein levels

**Fig 2:**
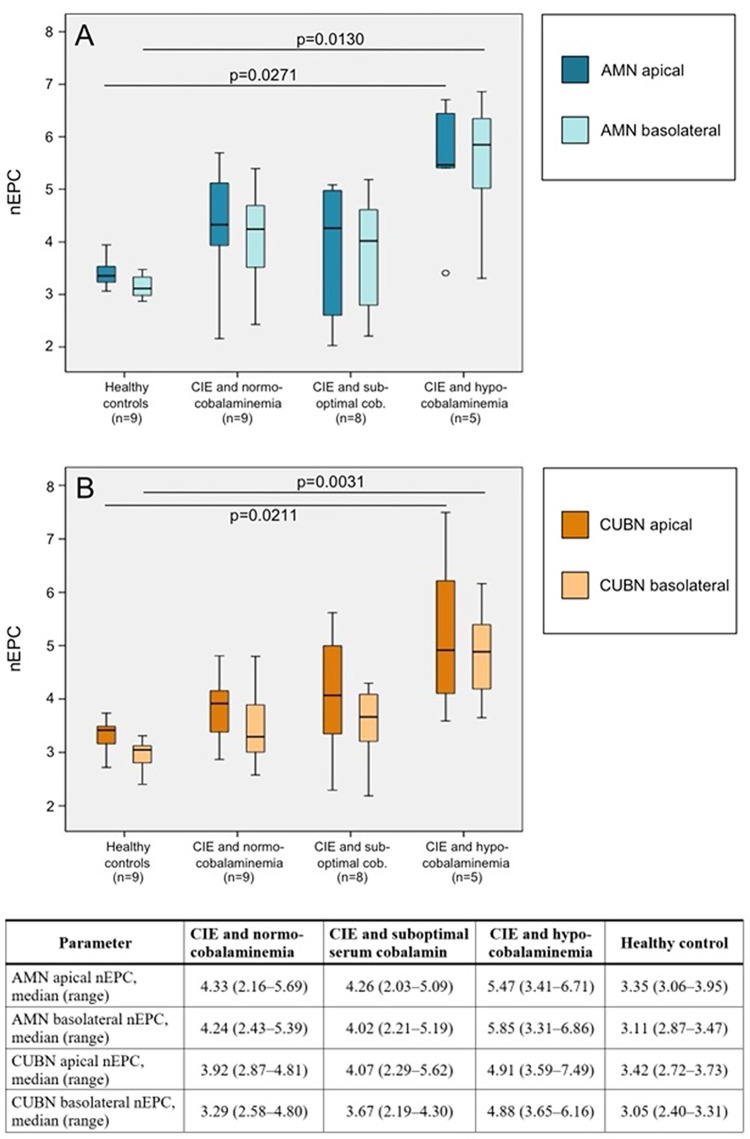
Ileal cobalamin receptor expression in canine chronic inflammatory enteropathy (CIE) and in health. Quantitative confocal laser scanning microscopy analysis and comparison of (A) amnionless (AMN) and (B) cubilin (CUBN) in the apical and basolateral compartment of the intestinal epithelial cells among the four groups of dogs: healthy controls, and dogs with CIE that were either normocobalaminemic (serum cobalamin concentration 400–908 ng/L), have a suboptimal serum cobalamin status (251–400 ng/L) or are hypocobalaminemic (serum cobalamin concentration ≤250 ng/L). Cobalamin receptor expression was determined by computation of normalized epithelial photon counts (nEPC). Data showed increased levels of both receptor subunits in dogs with CIE, particularly hypocobalaminemic dogs, compared to healthy controls. Boxplots mark the median, quartiles, and range; outliers are indicated by a circle.

Immunofluorescence analysis of cobalamin receptor abundance in the apical and basolateral compartment of the ileal epithelial cells revealed significantly different levels of both receptor subunits among the four groups of dogs (AMN apical nEPC: *P* = 0.0288; AMN basolateral nEPC: *P* = 0.0188; CUBN apical nEPC: *P* = 0.0264; CUBN basolateral nEPC: *P* = 0.0052; Fig 2). Receptor levels were significantly increased in severely hypocobalaminemic dogs with CIE compared to healthy controls (AMN apical nEPC: *P* = 0.0271; AMN basolateral nEPC: *P* = 0.0130; CUBN apical nEPC: *P* = 0.0211; CUBN basolateral nEPC: *P* = 0.0031). There were strong to very strong positive correlations between the AMN and CUBN protein levels and among their levels in the apical and basolateral compartment of ileal epithelial cells (S3 Table).

### Correlation of ileal AMN and CUBN protein levels with patient characteristics, clinical and clinicopathologic variables, and histopathologic lesions

**Fig 3:**
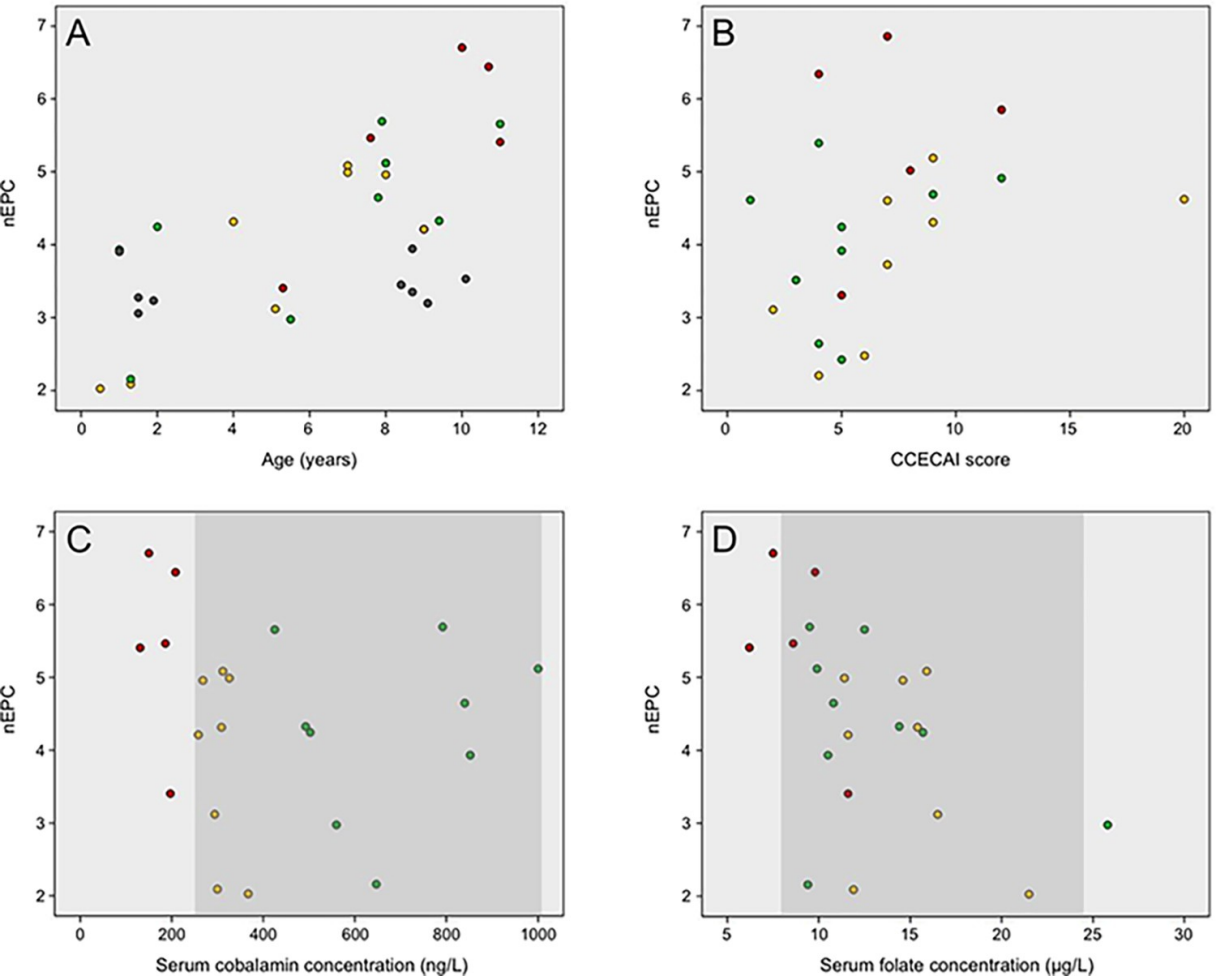
Correlation of ileal cobalamin receptor expression with clinical and clinicopathological patient variables. Groups of dogs are indicated by different colors: green = dogs with chronic inflammatory enteropathy (CIE) and normocobalaminemia, yellow = CIE dogs with suboptimal cobalamin status, and red = CIE dogs with severe hypocobalaminemia; and gray dots indicate the healthy control group. Each y-axis shows the corresponding normalized epithelial photon counts (nEPC). A: Correlation between apical amnionless (AMN) expression and the dogs’ age (*ρ* = 0.80, *P*<0.0001; including healthy controls: *ρ* = 0.61, *P* = 0.0003). B: Correlation between basolateral AMN expression and CCECAI scores (*ρ* = 0.42, *P* = 0.0491; only including dogs with CIE). C: No correlation was seen between the apical AMN expression and serum cobalamin concentrations (*ρ* = -0.23, *P* = 0.2963; only including CIE dogs). Dark gray area: reference interval for serum cobalamin concentration (251–908 ng/L; www.vetmed.tamu.edu/gilab). D: Correlation of the apical AMN expression with serum folate concentrations (*ρ* = -0.55, *P* = 0.0078, *P*_*corr*_ = 0.0312; only CIE dogs). Dark gray: reference interval for serum folate concentration (7.7–24.4 μg/L, www.vetmed.tamu.edu/gilab).

Results for the correlation analysis of ileal AMN and CUBN protein levels with clinical, clinicopathologic, and histologic data are summarized in Table 2. Ileal cobalamin receptor levels were moderately positively correlated with age (AMN apical nEPC: *ρ* = 0.80, *P*<0.0001; including healthy controls: *ρ* = 0.61, *P* = 0.0003, Fig 3A; AMN basolateral nEPC: *ρ* = 0.72, *P* = 0.0002; including healthy controls: *ρ* = 0.53, *P* = 0.0023) and the CCECAI score (AMN basolateral nEPC *ρ* = 0.42, *P* = 0.0491; Fig 3B).

**Table 2:** Correlation (Spearman *ρ*) of ileal cobalamin receptor protein expression with clinical, clinicopathologic, and histologic variables in dogs with CIE (n = 22).

Parameter correlated with	Apical AMN expression	Basolateral AMN expression	Apical CUBN expression	Basolateral CUBN expression
***Patient characteristics***				
Age	**0.80** (*P<*0.0001)	**0.72** (*P* = 0.0002)	0.41	**0.452** (*P* = 0.041)
***Clinical parameters***				
CCECAI score	0.39	**0.42** (*P* = 0.0491)	0.42	0.34
***Clinicopathologic parameters***				
Serum cobalamin concentration	-0.23	-0.28	-0.31	-0.41
Serum folate concentration	**-0.55** (*P*_*corr*_ = 0.0312)	**-0.57** (*P*_*corr*_ = 0.0212)	-0.22	**-0.48** (*P*_*corr*_>0.05)
Serum CRP concentration	0.36	0.40	0.14	0.39
Serum albumin concentration	-0.37	-0.37	-0.19	-0.34
***Histopathologic parameters***				
Ileum (composite Histo-score)	0.01	0.07	0.17	0.07
*Morphologic criteria*				
Villus stunting	0.01	-0.14	0.30	0.03
Epithelial injury	-0.30	-0.30	0.06	0.12
Crypt distension	0.11	0.17	0.17	0.38
Lacteal dilation	0.31	**0.47** (*P*_*corr*_>0.05)	0.19	0.25
Mucosal fibrosis	0.11	-0.06	-0.20	-0.36
*Inflammatory criteria*				
Intraepithelial lymphocytes	0.05	0.13	0.19	0.25
Lamina propria LPC	0.07	0.14	-0.05	-0.12
Lamina propria eosinophils	**-0.46** (*P*_*corr*_>0.05)	-0.39	-0.41	**-0.45** (*P*_*corr*_>0.05)
Lamina propria neutrophils	-0.21	-0.33	0.04	-0.08
Lamina propria ΜΦ	0.05	-0.11	0.26	0.11

CCECAI: Canine chronic enteropathy clinical activity index; CRP: C-reactive protein; LPC: Lymphocytes and plasma cells; ΜΦ: Macrophages. Values in bold font and highlighted in blue indicate significance at *P*<0.05. *P*_*corr*_: *P*-value after Holm-Bonferroni correction (n = 4 or 5).

There was no association between cobalamin receptor levels and serum cobalamin concentrations (Fig 3C). However, receptor levels were moderately inversely correlated with serum folate concentrations (AMN apical nEPC: *ρ* = -0.55, *P*_*corr*_ = 0.0312; Fig 3D; AMN basolateral nEPC: *ρ* = -0.57, *P*_*corr*_ = 0.0212). Only on univariate analysis were cobalamin receptor levels moderately positively correlated with the severity of lacteal dilation in the ileum (AMN basolateral nEPC: *ρ* = 0.47, *P* = 0.0279, *P*_*corr*_>0.05) and inversely associated with the severity of eosinophilic infiltration in the ileal mucosa (AMN apical nEPC: *ρ* = -0.46, *P* = 0.0319, *P*_*corr*_>0.05; CUBN basolateral nEPC: *ρ* = -0.45, *P* = 0.0426, *P*_*corr*_>0.05; Table 2). Ileal cobalamin receptor levels did not correlate with any other patient characteristics, histopathologic criteria in the duodenum or ileum, or biomarkers measured. Given the different sex distribution among the groups of dogs, a potential effect on AMN and CUBN protein levels was tested. No significant interaction was identified between group (CIE *vs*. healthy control) and sex (male vs. female) (all *P*<0.05; Table 3), and the effects test for the model showed the only significant determinant of AMN and CUBN levels to be the assigned group.

**Table 3:** Effects tests for the variables “group” and “sex” as well as their interaction term on ileal AMN and CUBN protein levels. Shown are the *P*-values for the individual effects.

Outcome variable	*P* (CIE vs. healthy)	*P* (male vs. female)	*P* (interaction)
AMN (apical)	0.0603	0.4398	0.1475
AMN (basolateral)	**0.0268**	0.4770	0.1956
CUBN (apical)	**0.0306**	0.5721	0.6676
CUBN (basolateral)	**0.0128**	0.5228	0.5801

### Mucosal gene expression of the ileal cobalamin receptor and inflammatory mediators

**Fig 4:**
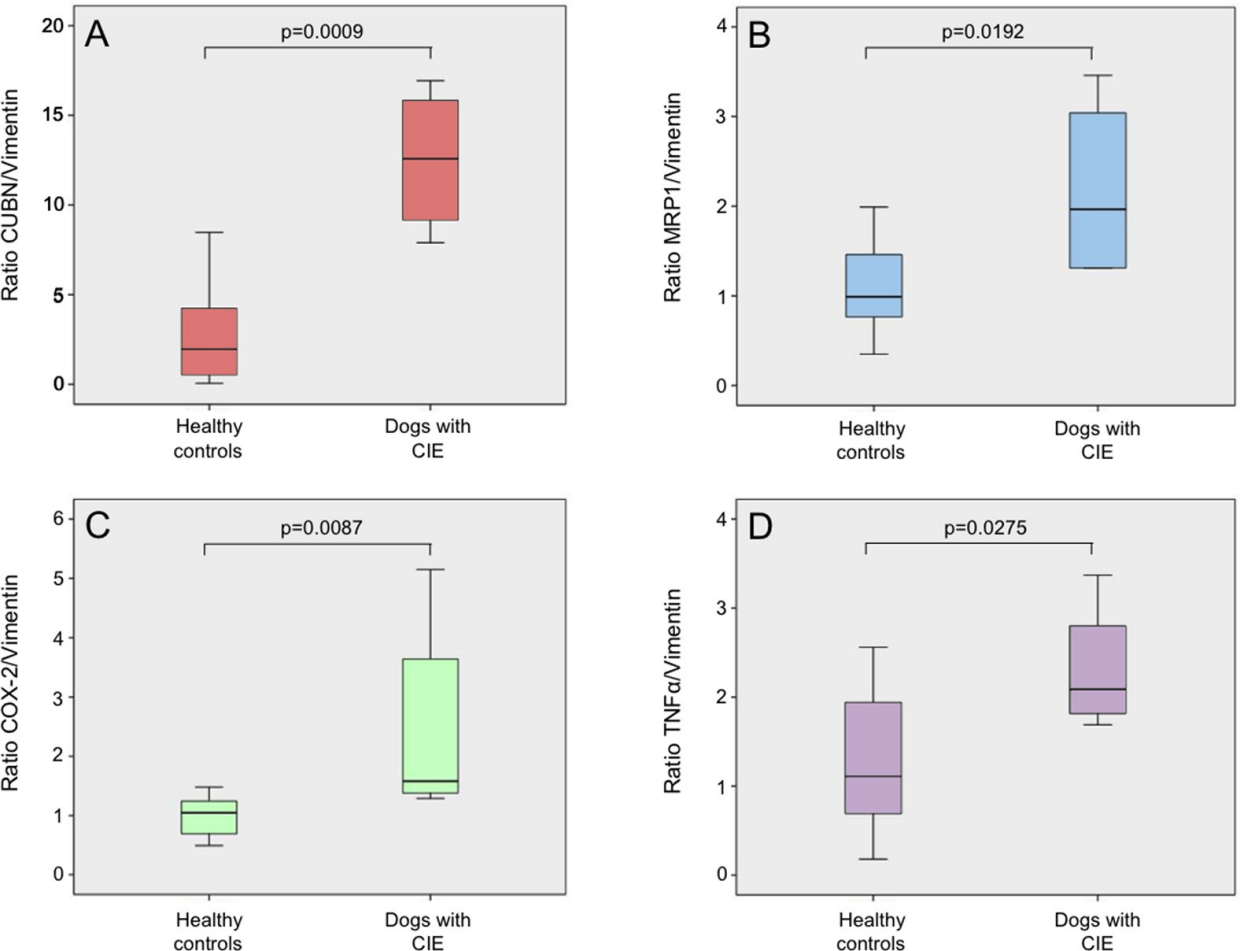
Ileal mucosal mRNA expression levels of the cobalamin receptor and inflammatory mediators. Comparison of mucosal mRNA expression for (A) cubilin (*CUBN)*, (B) multidrug-resistance-related protein 1 (*MRP1)*, (C) cyclooxygenase-2 (*COX-2)*, and (D) and tumor necrosis factor-alpha *(TNFA)* between healthy control dogs (n = 11) and dogs with chronic inflammatory enteropathy (CIE) (n = 5). Expression of each target gene, normalized against the validated housekeeping genes *SGLT1*, *HPRT1*, *SDHA*, and *RPL8*, is expressed relative to vimentin (*VIM*) to account for differing connective tissue contents of the samples. Boxplots mark the median, quartiles, and range; outliers are not shown (A: mild outliers [inner Tukey fences] for 1 control dog with *CUBN/VIM* = 8.5; and 1 CIE dog with *CUBN/VIM* = 31.6. B: extreme outlier [outer Tukey fences] for 1 IBD dog: *MRP1/VIM* = 15.9. C: extreme outlier for 1 control dog: *COX-2/VIM* = 3.0. D: extreme outlier for 1 IBD dog: *TNFA/VIM* = 9.5).

mRNA expression of *CUBN* was significantly increased in dogs with CIE (median: 14.7, range: 7.9–31.6 for *CUBN/VIM* ratio) compared to healthy controls (median: 1.3, range: 0.1–8.5 for *CUBN/VIM* ratio) (*P* = 0.0009; Fig 4A). Similarly, *MRP1* was significantly higher in CIE dogs (median: 2.6, range: 1.3–15.9 for *MRP1/VIM* ratio) than healthy controls (median: 1.0, range: 0.4–2.0 for *MRP1/VIM* ratio; *P* = 0.0192; Fig 4B).

Increased expression of *COX-2* (*P* = 0.0087; Fig 4C) and *TNFA* (*P* = 0.0275; Fig 4D) was also observed in dogs with CIE (median: 1.6, range: 1.3–5.2 for *COX-2/VIM* ratio; median: 2.2; range: 1.7–9.5 for *TNFA/VIM* ratio) compared to the control group (median: 1.1, range: 0.5–3.0 for *COX-2/VIM* ratio; median: 1.1, range: 0.2–2.6 for *TNFA/VIM* ratio).

## Discussion

**Fig 5:**
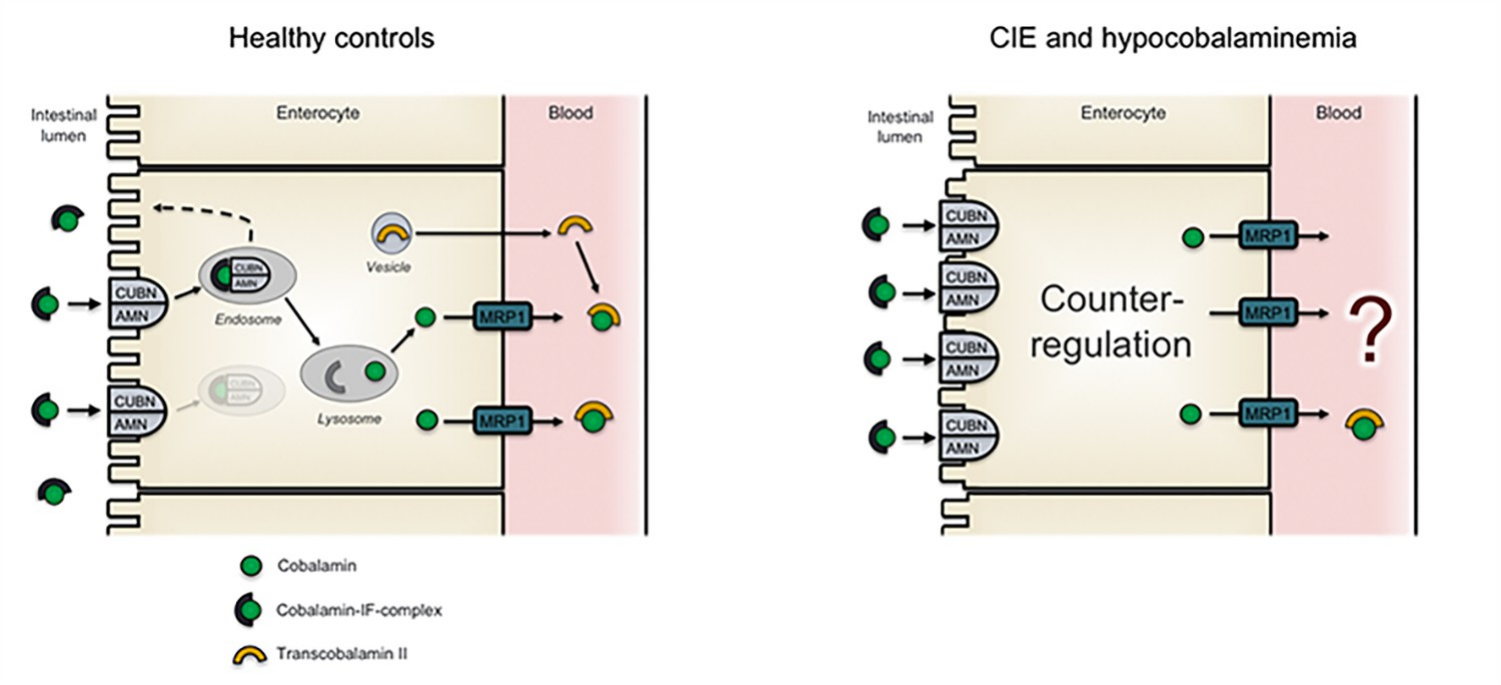
Compensatory upregulation of the cobalamin transport capacity in dogs with chronic inflammatory enteropathy (CIE). Compared to health (left image), hypocobalaminemic dogs with CIE have significant upregulation of the ileal cobalamin receptor reserve, likely as a compensatory mechanism to counteract hypocobalaminemia.

This study documented increased ileal cobalamin receptor abundances in dogs with CIE based on protein and mRNA expression levels. Typically, the excessive immune response of the intestinal mucosa in dogs with CIE is characterized by infiltrating inflammatory cells (mostly lymphocytes and plasma cells) and structural lesions (e.g., villus stunting, lacteal dilation) [52]. This, together with the finding of reduced cobalamin receptor levels in pigs with proliferative ileitis [56], led to the previous assumption of a downregulated expression of the cobalamin receptor in the ileum of dogs with CIE as a consequence of severe and/or chronic inflammation [42]. Our findings disprove this long-held assumption. Contrary to past beliefs, the ileal mucosal protein levels of both receptor subunits, CUBN and AMN, as well as mRNA expression of *CUBN* and *MRP1* were significantly increased in hypocobalaminemic dogs with CIE compared to healthy controls. We interpret this finding as a compensatory upregulation of the transport capacity for cobalamin to outbalance hypocobalaminemia (Fig 5). Our findings also do not suggest significant changes in the intracellular location or basolateral trafficking of the ileal epithelial cobalamin receptor in dogs with IBD.

The exact mechanisms involved in the severe reduction of the circulating cobalamin pool in canine CIE remain unknown. It is possible that the systemic disease process is associated with an increased demand for cobalamin (e.g., for tissue regeneration). An alternative reason could be a decreased ileal uptake of cobalamin due to intestinal dysbiosis and increased abundances of *Bacteroides* spp. carrying a competitive advantage of high-affinity cobalamin binding [57]. Receptor dysfunction or a reduction in total ileal epithelial mass resulting from the inflammatory lesions and/or epithelial damage are also possible causes. Other possible explanations are increased urinary losses of cobalamin associated with reduced reabsorption after glomerular filtration [34,58], decreased intestinal reabsorption (enterohepatic circulation) of biliary cobalamin [59,60], or increased abundances of luminal and/or endogenously synthesized cobalamin analogues competing with cobalamin metabolism [61,62]. Compensatory mechanisms of ileal epithelial cobalamin receptor upregulation would be expected in any dog with a suboptimal cobalamin supply, to allow increased cobalamin absorption and basolateral efflux. Altered cobalamin receptor recycling to increase the efficiency of cobalamin uptake might also play a role [57].

Our results showed no association between ileal cobalamin receptor protein levels and serum cobalamin concentrations, but an inverse correlation with serum folate concentrations. In addition to this relationship being solely explained by the presence of intestinal dysbiosis, it could indicate that some dogs already have a suboptimal intracellular cobalamin supply or intracellular cobalamin deficiency resulting in a functional folate deficiency, despite (still) having normal serum cobalamin concentrations because the intracellular metabolic pathways of cobalamin and folate metabolism are interconnected [18]. Given that cobalamin-dependent metabolic reactions are localized intracellularly, the concentration of cobalamin in serum does not necessarily reflect the cobalamin status on the cellular level. Several studies showed that some dogs with CIE and serum cobalamin concentrations within (normocobalaminemia) or at the lower end of the reference interval (suboptimal serum cobalamin status) have increased serum MMA concentrations, indicating cellular cobalamin deficiency [63,64]. The increased serum MMA concentrations will decrease (i.e., normalize) again in these dogs after successful supplementation with cobalamin [64]. In human medicine, eight cobalamin complementation groups (cblA to cblH) are described, which are important for the synthesis of adenosylcobalamin (one of the biologically active coenzyme forms of cobalamin). Patients with inborn errors show signs of cobalamin deficiency and cobalamin-responsive methylmalonic acidemia [65,66]. In veterinary medicine, no comparable studies are available.

In our study, an older age (>7 years) and more severe clinical disease (CCECAI score ≥9) correlated with higher cobalamin receptor protein levels in the CIE group of dogs, likely reflecting higher demands for cobalamin in older patients. It might be argued that the upregulation of cobalamin transport proteins is not caused by CIE and that the patient age and higher prevalence of CIE in older dogs might confound the data [9,10]. Older dogs with CIE also show more often disease progression and lack of full disease remission after treatment [10,67]. An experimental study revealed decreased ileal AMN expression levels in aging rats [34], which would contradict that the ileal cobalamin receptor upregulation observed was merely age-related. However, species differences also have to be considered. Also, increased AMN levels precede the development of cobalamin deficiency [34] which is consistent with our findings. While this upregulation might be initiated by systemic signaling due to the metabolic imbalances caused by cobalamin deficiency, it might also be directly elicited by the inflammatory stimulus in the epithelium.

Cobalamin receptor levels in the ileal mucosa were not correlated with histopathologic lesion criteria on multivariate analyses. A moderate positive correlation with the severity of lacteal dilation and an inverse association with the severity of eosinophilic infiltration in the ileum were only detected on univariate analysis. While histologic lesions may not determine cobalamin uptake in the ileum, most dogs included in our study had only mild histologic changes. The severity of histologic changes is also not associated with clinical outcomes in canine CIE [10,68] but histopathological lesions in the ileum correlated with low serum cobalamin concentrations (<200 ng/L) [41].

Structural and inflammatory changes in the ileal mucosa and the assumption that cobalamin uptake might be impaired due to CIE resulted in the traditional recommendation to administer supplemental cobalamin strictly via the parenteral route, either subcutaneously (cyanocobalamin) or intramuscularly (hydroxocobalamin) [39,63,64,69]. Recent studies proved that cobalamin supplementation can be effective with oral administration in dogs with gastrointestinal disease [64,70] but individual responses can vary [71]. Transporting cobalamin from the intestinal lumen across the enterocytes and into the bloodstream requires efficient uptake mechanisms, which entail cubam-mediated endocytosis of the cobalamin-IF complex [33], intracellular vectorial transport of cobalamin, and, in addition to its utilization by the enterocyte (approximately 20%), basolateral trafficking and extrusion (about 80%) via MRP1 [36,57].

Contrary to our findings, only apical epithelial expression of the cobalamin receptor in microvillus pits, vesicles, and endoplasmic reticulum of enterocytes was previously shown [68]. We found the cobalamin receptor to also be expressed in the basolateral compartment of ileal enterocytes and a strong correlation between apical and basolateral receptor levels. Differences in tissue sampling, antibody, and technique employed (transmission electron microscopy vs. CLSM) might explain that discrepancy. Similar to our results, AMN- and CUBN-positive immunofluorescence staining was not limited to the apical enterocyte compartment in the human terminal ileum [72]. It remains speculative whether this reflects remnants of internalized apical receptors or if cubam has a role in the basolateral extrusion of cobalamin. However, this study is the first to quantify protein levels of the cobalamin receptor subunits in ileal enterocytes and in dogs. None of these appear to be the primary determinant for hypocobalaminemia in canine CIE, but paracellular absorption may also partially contribute to receptor-mediated uptake [73].

Intestinal *TNFA* and *COX-2* mRNA levels were altered in dogs with CIE. This agrees with a previous study in dogs [48], but contrasts with a meta-analysis revealing heterogeneous intestinal cytokine signatures (and varying *TNFA* mRNA levels) in canine IBD [74] and a lack of proinflammatory cytokine upregulation in other canine CIE studies [75,76]. Pro- and anti-inflammatory cytokines are essential for mucosal homeostasis [74], and differences in age, stage of growth, and nutritional status can affect that balance [77] and may explain the varying results for inflammatory cytokines in CIE [74].

We acknowledge a few limitations of this study. There was a difference in breed heterogeneity between the study groups. Dogs included in the control group were all purpose-bred hound dogs, whereas the CIE group represented various dog breeds. However, disorders of cobalamin metabolism are not known in hound dogs, and an effect of age and (chronic) disease on serum cobalamin concentrations appears more likely. However, all groups included a spectrum of younger and older dogs but an effect of differences in husbandry, diet, and/or other environmental and lifestyle factors on the results of this study can also not be excluded. Also, the total epithelial (and thus, total cobalamin-receptor) mass cannot be evaluated using the techniques employed in this study, and the compensatory upregulation of ileal cobalamin receptor levels may equal the overall reduction of functional epithelium (e.g., with villus stunting and/or fusion). With an unchanged overall capacity for cobalamin absorption, increased demands for cobalamin could also contribute to serum cobalamin deficiency states. Further evaluating the exact localization of the receptor and its functionality is also warranted. For immunofluorescence analysis, endoscopic tissue biopsy samples of the canine ileum were fixed in formalin, paraffin-embedded, and sectioned at 7 μm thickness. As can be expected with (even good-quality and well-oriented) endoscopic tissue biopsies, some blocks or individual villi of the same biopsy were to some degree tangentially and not perpendicularly cut. These tissue samples are routinely re-oriented prior to embedding, but due to the nature of an endoscopic biopsy, not all villi in this optimized tissue specimen will be exactly perpendicularly oriented and cut. Lastly, the cobalamin status of the dogs was determined based on the serum cobalamin concentration, and serum HCY and/or MMA concentrations may have been useful additional markers to assess the intracellular availability of cobalamin. Available quantities of serum samples were limited, and a retrospective analysis of these metabolites in serum was not possible. Measuring serum cobalamin, HCY, MMA, and holotranscobalamin together with a factor normalizing for the patient’s age, is used to assess the cobalamin status in people [78]. In small animal practice, measurement of serum cobalamin concentration, often combined with serum folate concentration, is presently routinely performed in patients with suspected cobalamin deficiency [79,80]. To assess the cobalamin status more accurately absent any biomarkers of cobalamin metabolism or transport, the group of dogs with CIE was stratified into 3 subgroups based on serum cobalamin status instead of a dichotomous classification. This was important as the group of dogs with a suboptimal serum cobalamin concentration likely included some dogs with a serum cobalamin concentration in the lower range of the reference interval and possible intracellular cobalamin deficiency. Fedosov et al. [78] proposed an evaluation of the body’s cobalamin status when additional biomarkers (i.e., MMA, HCY, or holotranscobalamin concentrations) are not available from a patient by correcting for that missing data point. Specifically, assessment of the cobalamin status is recommended to be adjusted when the serum folate concentration is <10 nmol/L (4.41 μg/L), and calculations include the HCY concentration [78]. However, serum folate concentration in CIE dogs in our study ranged from 6.2–25.8 μg/L. Thus, correcting the assessment of the cobalamin status by applying this method was expected to have negligible effects (if any) on the assignment of individual dogs in the CIE group to different subgroups based on their cobalamin status.

In conclusion, an increased abundance of the cobalamin receptor subunits CUBN and AMN, and the basolateral efflux transporter MRP1, was documented in the ileal epithelium of hypocobalaminemic dogs with CIE, especially with older age and more severe clinical signs of GI disease. This challenges the previous theory that dogs with CIE develop hypocobalaminemia due to intestinal epithelial cobalamin receptor downregulation. In contrast, we showed a counter-regulation of the ileal cobalamin receptor, which we propose aims to meet increased demands (and/or sequestration or renal/biliary loss) of cobalamin due to the chronic state of inflammation. Functional studies and evaluation of ileal mucosal megalin co-localization need to further clarify the intestinal epithelial cobalamin transport mechanisms.

## Supporting information

S1 Fig Confocal laser scanning microscopy of the CUBN subunit. Immunofluorescent staining of the cobalamin receptor subunit CUBN in the ileum of (A) healthy control dog and (D) hypocobalaminemic dog with CIE. Secondary antibody control staining (B&E) and staining with blocking peptide (C&F) demonstrate the specificity of the antibody for CUBN.
(DOCX)

S2 Fig Confocal laser scanning microscopy of the AMN subunit in ileal endoscopic biopsies from dogs. A: Immunofluorescent staining of the cobalamin receptor subunit AMN in the ileum of a hypocobalaminemic dog with chronic inflammatory enteropathy. B and C: Staining controls using a commercial blocking peptide (B) or a secondary antibody-only control (C) are also shown. The AMN subunit is stained in green; nuclei are counter-stained in blue using DAPI (diamidine phenylindole). Images were processed with dye separation and deconvolution.
(DOCX)

S3 Fig Western blot analysis for the AMN antibody without and with preincubation with the corresponding blocking peptide. Ileum epithelium from 4 different dogs (test samples; lanes 2–5 and lanes 9–12), as well as murine ileum (lanes 6 and 13) and kidney (lanes 7 and 14) as positive controls, were homogenized in RIPA buffer, separated by SDS-PAGE, and were blotted onto a nitrocellulose membrane. After blocking, the membranes were incubated either with anti-AMN (at a 1:500 dilution; left side of the blot, lanes 2–7) or with anti-AMN + blocking peptide (at a 1:500 dilution + 1:50 dilution, preincubated for 12h at 4°C; right side of the blot, lanes 9–14) over night. The signal was detected using an HRP-coupled secondary antibody and enhanced chemiluminescence. A specific band for AMN was observed at approximately 37 kDa (blue arrow pointing to the left side of the blue box) and was absent after preincubation of the antibody with the peptide (right side of the blue box), supporting the competitive binding of the AMN-antibody with the blocking peptide.
(DOCX)

S1 Table Primers used for qPCR analysis. (DOCX)

S2 Table Characteristics of all CIE dogs (n = 5) included in the qPCR analysis. (DOCX)

S3 Table Correlations between the two cobalamin receptor subunits AMN and CUBN in the apical and basolateral compartments of ileal enterocytes in dogs with CIE (n = 22). Values in bold font and highlighted in blue indicate significance at *P*<0.05.
(DOCX)
