# Supplementary material for: Expression of the cobalamin transporters cubam and MRP1 in the canine ileum–Upregulation in chronic inflammatory enteropathy
Source: PLoS One. 2024 Jan 11;19(1):e0296024. doi: 10.1371/journal.pone.0296024 (PMC10783779; doi:10.1371/journal.pone.0296024)

**S1 Fig. Confocal laser scanning microscopy of the CUBN subunit.** Immunofluorescent staining of the cobalamin receptor subunit CUBN in the ileum of (A) healthy control dog and (D) hypocobalaminemic dog with CIE. Secondary antibody control staining (B&E) and staining with blocking peptide (C&F) demonstrate the specificity of the antibody for CUBN.


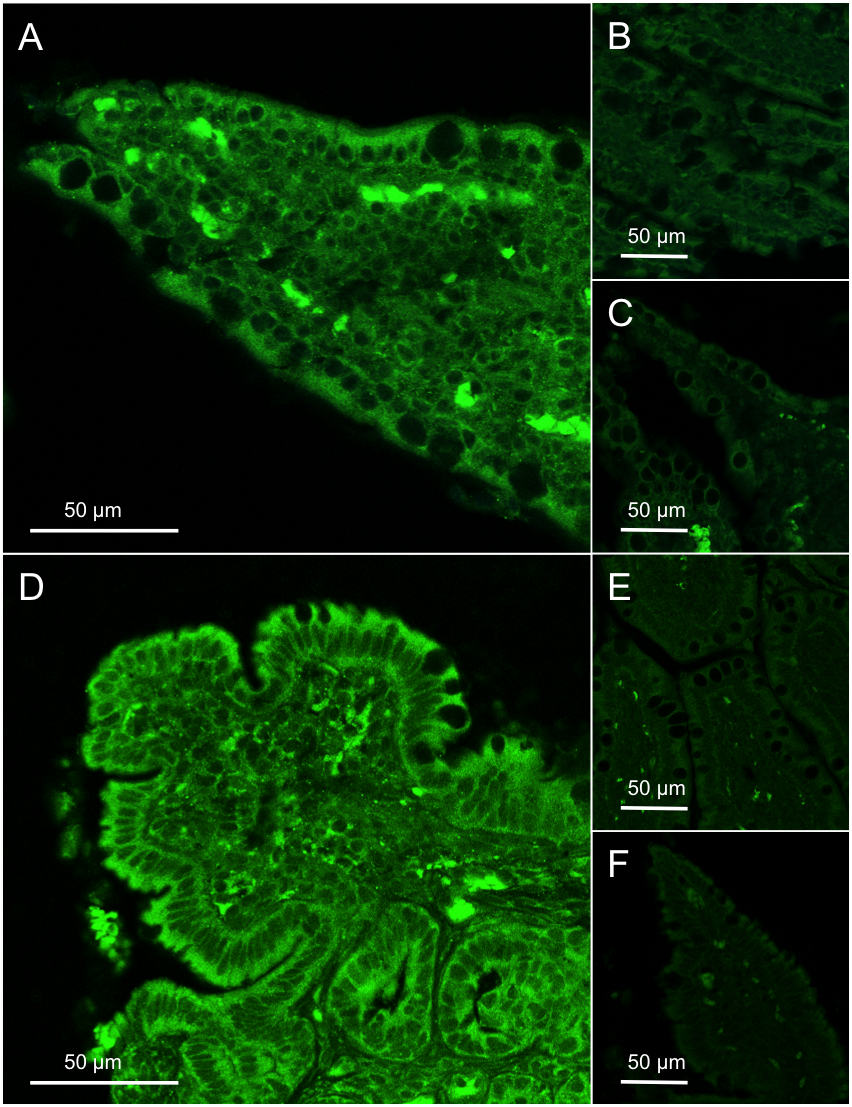

Supplement: S1 Fig — Immunofluorescent staining of the cobalamin receptor subunit CUBN in the ileum of (A) healthy control dog and (D) hypocobalaminemic dog with CIE. Secondary antibody control staining (B&E) and staining with blocking peptide (C&F) demonstrate the specificity of the antibody for CUBN. (DOCX) [file pone.0296024.s001.docx]
