# Supplementary material for: Expression of the cobalamin transporters cubam and MRP1 in the canine ileum–Upregulation in chronic inflammatory enteropathy
Source: PLoS One. 2024 Jan 11;19(1):e0296024. doi: 10.1371/journal.pone.0296024 (PMC10783779; doi:10.1371/journal.pone.0296024)

**S2 Fig.** **Confocal laser scanning microscopy of the AMN subunit in ileal endoscopic biopsies from dogs.** A: Immunofluorescent staining of the cobalamin receptor subunit AMN in the ileum of a hypocobalaminemic dog with chronic inflammatory enteropathy. B and C: Staining controls using a commercial blocking peptide (B) or a secondary antibody-only control (C) are also shown. The AMN subunit is stained in green; nuclei are counter-stained in blue using DAPI (diamidine phenylindole). Images were processed with dye separation and deconvolution.


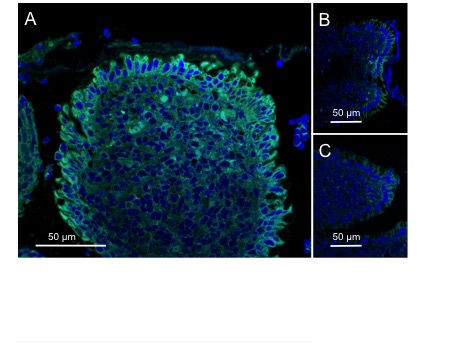

Supplement: S2 Fig — A: Immunofluorescent staining of the cobalamin receptor subunit AMN in the ileum of a hypocobalaminemic dog with chronic inflammatory enteropathy. B and C: Staining controls using a commercial blocking peptide (B) or a secondary antibody-only control (C) are also shown. The AMN subunit is stained in green; nuclei are counter-stained in blue using DAPI (diamidine phenylindole). Images were processed with dye separation and deconvolution. (DOCX) [file pone.0296024.s002.docx]
