# Supplementary material for: Expression of the cobalamin transporters cubam and MRP1 in the canine ileum–Upregulation in chronic inflammatory enteropathy
Source: PLoS One. 2024 Jan 11;19(1):e0296024. doi: 10.1371/journal.pone.0296024 (PMC10783779; doi:10.1371/journal.pone.0296024)

**S3 Fig.** **Western blot analysis for the AMN antibody without and with preincubation with the corresponding blocking peptide.** Ileum epithelium from 4 different dogs (test samples; lanes 2–5 and lanes 9–12), as well as murine ileum (lanes 6 and 13) and kidney (lanes 7 and 14) as positive controls, were homogenized in RIPA buffer, separated by SDS-PAGE, and were blotted onto a nitrocellulose membrane. After blocking, the membranes were incubated either with anti-AMN (at a 1:500 dilution; left side of the blot, lanes 2–7) or with anti-AMN + blocking peptide (at a 1:500 dilution + 1:50 dilution, preincubated for 12h at 4°C; right side of the blot, lanes 9–14) over night. The signal was detected using an HRP-coupled secondary antibody and enhanced chemiluminescence. A specific band for AMN was observed at approximately 37 kDa (blue arrow pointing to the left side of the blue box) and was absent after preincubation of the antibody with the peptide (right side of the blue box), supporting the competitive binding of the AMN-antibody with the blocking peptide.


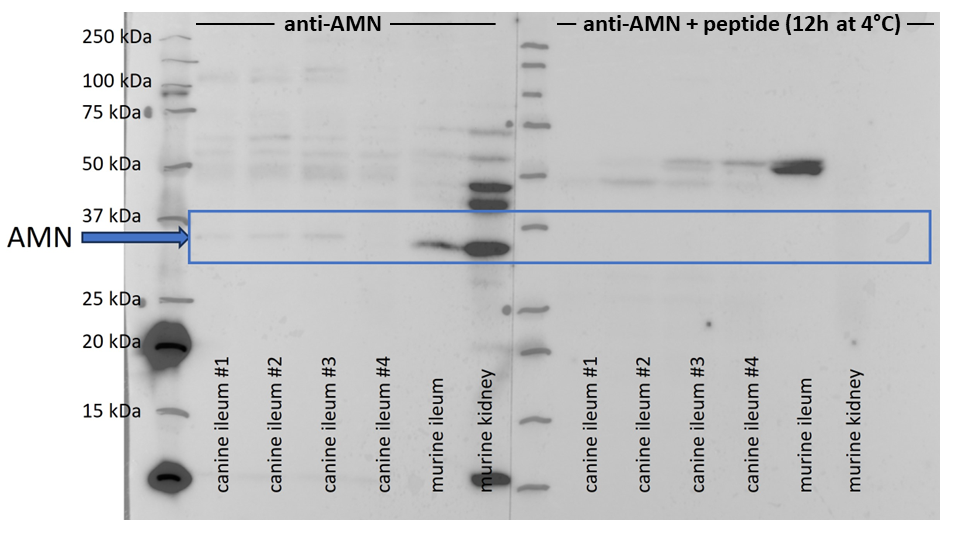

Supplement: S3 Fig — Ileum epithelium from 4 different dogs (test samples; lanes 2–5 and lanes 9–12), as well as murine ileum (lanes 6 and 13) and kidney (lanes 7 and 14) as positive controls, were homogenized in RIPA buffer, separated by SDS-PAGE, and were blotted onto a nitrocellulose membrane. After blocking, the membranes were incubated either with anti-AMN (at a 1:500 dilution; left side of the blot, lanes 2–7) or with anti-AMN + blocking peptide (at a 1:500 dilution + 1:50 dilution, preincubated for 12h at 4°C; right side of the blot, lanes 9–14) over night. The signal was detected using an HRP-coupled secondary antibody and enhanced chemiluminescence. A specific band for AMN was observed at approximately 37 kDa (blue arrow pointing to the left side of the blue box) and was absent after preincubation of the antibody with the peptide (right side of the blue box), supporting the competitive binding of the AMN-antibody with the blocking peptide. (DOCX) [file pone.0296024.s003.docx]
