# Supplementary material for: Expression of the cobalamin transporters cubam and MRP1 in the canine ileum–Upregulation in chronic inflammatory enteropathy
Source: PLoS One. 2024 Jan 11;19(1):e0296024. doi: 10.1371/journal.pone.0296024 (PMC10783779; doi:10.1371/journal.pone.0296024)
Supplement: S1 Table — (DOCX) [file pone.0296024.s004.docx]

**S1 Table. Primers used for qPCR analysis.**

| **Gene name** | **GenBank accession number** | **Forward primer (5’ – 3’)** | **Reverse primer (3’ – 5’)** | **Annealing temp. (°C)** |
| --- | --- | --- | --- | --- |
| *CUBN* | NM_001003148.1 | CTGGAAGACCTGTCACCGTC | GCATCCGGTCCATCGTAGAG | 61 |
| *MRP1* | NM_001002971.1 | GGGCTCCCTGTTCAATGTCA | GCCACGTAAAACCTCTGCAC | 60 |
| *SGLT1* | NM_001007141.1 | ACCGTCCCCTCGGAATGTAA | TCAGACCTCGCAGTCCATCA | 59 |
| *VIM* | NM_001287023.1 | GGATGCACTCAAAGGGACTAATG | GTCTTGGTAGTTAGCAGCTTCG | 60 |
| *TNF-α* | NM_001003244.4 | CTGCACTTTGGAGTGATCGG | GGGTTCGAGAAGATGATTTGACT | 58 |
| *COX-2* | NM_001003354.1 | AGCTTCGATTGACCAGAGCA | CAGCCATTTCCTTCTCTCCTGT | 59 |
| *HPRT1* | NM_001003357.2 | CCCAGCGTCGTGATTAGTGA | CACTTTTTCCAAATCCTCAGCGT | 60 |
| *SDHA* | XM_535807.6 | TCCGTGTGGGAAGTGTGTTA | GTGTTCCAGACCATTCCTCG | 57 |
| *RPL8* | XM_532360.2 | AACACATCGGCAAACCCTCT | TCCTGCACAGTCTTGGTTCC | 58 |
