# Supplementary material for: Expression of the cobalamin transporters cubam and MRP1 in the canine ileum–Upregulation in chronic inflammatory enteropathy
Source: PLoS One. 2024 Jan 11;19(1):e0296024. doi: 10.1371/journal.pone.0296024 (PMC10783779; doi:10.1371/journal.pone.0296024)
Supplement: S2 Table — (DOCX) [file pone.0296024.s005.docx]

**S2 Table. Characteristics of all CIE dogs (n = 5) included in the qPCR analysis.**

| **Patient characteristic** | **Normocobalaminemia** | **Hypocobalaminemia** |
| --- | --- | --- |
| n | 2^†^ | 3^$^ |
| Age in years, median | 7.3 | 10.1 |
| Sex, male (neutered) / female (spayed) | 2 (2) / 0 (0) | 2 (2) / 1 (0) |
| Body weight in kg, median | 21.9 | 31.0 |
| ***Clinical parameters*** | | |
| CCECAI score, median | 6 | 8 |
| ***Clinicopathologic parameters*** | | |
| Serum cobalamin in ng/L, median | 391 | 132 |
| Serum folate in ng/L, median | 14.9 | 8.9 |
| Serum albumin in g/L, median | 32 | 29 |
| CCECAI: canine chronic enteropathy clinical activity index; CRP: C-reactive protein; IQR: interquartile range. ^†^1 of the dogs was also included in the CLSM analysis; ^$^2 of the dogs were also included in the CLSM analysis. | | |
