# Supplementary material for: Expression of the cobalamin transporters cubam and MRP1 in the canine ileum–Upregulation in chronic inflammatory enteropathy
Source: PLoS One. 2024 Jan 11;19(1):e0296024. doi: 10.1371/journal.pone.0296024 (PMC10783779; doi:10.1371/journal.pone.0296024)
Supplement: S3 Table — Values in bold font and highlighted in blue indicate significance at P<0.05. (DOCX) [file pone.0296024.s006.docx]

**S3 Table. Correlations between the two cobalamin receptor subunits AMN and CUBN in the apical and basolateral compartments of ileal enterocytes in dogs with CIE (n=22).** Values in bold font and highlighted in blue indicate significance at *P*<0.05.

|  | **Correlation *(Spearman* *ρ)*** |
| --- | --- |
| apical AMN - apical CUBN | 0.621 |
| apical AMN - basal AMN | **0.955** |
| apical AMN - basal CUBN | **0.669** |
| apical CUBN - basal AMN | **0.652** |
| apical CUBN - basal CUBN | 0.878 |
| basal CUBN - basal AMN | 0.754 |
